# Waning of SARS-CoV-2 IgG antibodies after vaccination: first results from the CoMoLo follow-up 2021

**DOI:** 10.1093/eurpub/ckac129.048

**Published:** 2022-10-25

**Authors:** C Kußmaul, A Schaffrath Rosario, J Allen, M Lange, C Koschollek, S Haller, M Schlaud, RKI CoMoLo Study Team

**Affiliations:** 1 Department of Epidemiology and Health Monitoring, Robert Koch Institute, Berlin, Germany; 2 Department of Infectious Disease Epidemiology, Robert Koch Institute, Berlin, Germany

## Abstract

**Background:**

In 2020, the study “Corona-Monitoring Lokal” (CoMoLo) assessed seroprevalences of SARS-CoV-2 IgG antibodies in four study locations that were particularly affected by outbreaks in the early stages of the pandemic in Germany. One of the objectives of the 2021 follow-up was to examine the development of immunological parameters over time, including the extent of IgG antibody waning after vaccination.

**Methods:**

Venous blood specimens were collected from a sample of initial study participants over a 2-week period between May and October 2021, with an oversampling of seropositive or previously infected individuals. Levels of IgG antibodies to the SARS-CoV-2 spike protein were measured from serum using Anti-SARS-CoV-2-QuantiVac-ELISA (IgG) tests by Euroimmun. Information on SARS-CoV-2 vaccinations or known infections was collected via online questionnaires or telephone interviews.

**Results:**

A total of 3328 participants (74% response) gave blood specimens for this follow-up study, with questionnaire information available for 2843 (85%) of these. Preliminary analyses suggest that in participants who had received two doses of a vaccine more than 3 weeks before giving blood (n = 1583), IgG levels decreased exponentially by about 9.8% (95%CI 9.1% - 10.4%) with each additional week since the last dose, when controlling for age, sex, and type of vaccine. There was evidence of this waning effect differing by vaccine type. Antibody levels also appear to decline with increasing age, according to preliminary results. Final results of the linear model used to assess the dynamics and predictive factors of antibody levels will be reported.

**Conclusions:**

This follow-up study will add evidence to an improved understanding of antibody waning after SARS-CoV-2 vaccination. Preliminary results are in line with international studies and may be helpful for discussions on potential benefits of further vaccinations in Germany.

**Key messages:**

• Antibodies induced by COVID-19 vaccination wane over time. The magnitude of this effect differs by vaccine type. Antibodies also decreased with increasing age.

• Our results may be helpful for discussions on potential benefits of further COVID-19 vaccinations in Germany.

